# Congenital hepatic fibrosis and related ciliopathies across the life course: current management, disease burden, and unmet needs in the era of genomic diagnosis and translational therapeutics

**DOI:** 10.3389/fped.2026.1928268

**Published:** 2026-09-03

**Authors:** Ao Wang, Jie He, Keliang Liu, Jinfeng Fu, Jianxun Chen

**Affiliations:** 1 Department of Pathology, Affiliated Hospital of Panzhihua University, Panzhihua, China; 2 Department of General Surgery, Affiliated Hospital of Panzhihua University, Panzhihua, China; 3 Minimally Invasive Endoscopy Center, Affiliated Hospital of Panzhihua University, Panzhihua, China; 4 Department of Hepatobiliary, Affiliated Hospital of Panzhihua University, Panzhihua, China

**Keywords:** burden, congenital hepatic fibrosis, current management options, ductal plate malformation, portal hypertension, unmet needs

## Abstract

Congenital hepatic fibrosis (CHF) is a rare autosomal recessive hepatobiliary developmental disorder characterised by extensive fibrosis of the portal tracts and ductal plate malformation, representing a significant cause of portal hypertension in children and adolescents. Despite its low incidence, the disease exhibits considerable clinical heterogeneity, ranging from asymptomatic presentations to severe complications of portal hypertension, thereby posing substantial diagnostic and management challenges. The long-term disease burden is profound, adversely affecting both quality of life and prognosis. Currently, clinical management focuses primarily on the surveillance and treatment of portal hypertensive complications, with a conspicuous absence of targeted therapies addressing the underlying disease processes. Furthermore, notable deficiencies persist in multidisciplinary coordination, individualised therapeutic strategies, and long-term follow-up frameworks. This review aims to comprehensively examine the pathophysiology, genetics, clinical manifestations, diagnostic approaches, complication management strategies, and therapeutic interventions pertaining to this condition. It systematically evaluates the associated disease burden and critically appraises the limitations of current diagnostic and treatment paradigms, as well as the unmet needs in clinical practice. Ultimately, this review seeks to provide critical insights and a theoretical framework to inform future research directions, optimise therapeutic regimens, and facilitate the development of integrated care models. Unlike previous reviews focusing mainly on pathology or case-based clinical presentation, this review emphasizes life-course management, patient burden, and translational gaps in the era of genomic diagnosis.

## 1 Introduction

Congenital hepatic fibrosis (CHF) is a rare autosomal recessive fibrocystic liver disease that originates from defective remodelling of the embryonic ductal plate, resulting in characteristic periportal fibrosis and intrahepatic bile duct abnormalities (1). The disease represents a core entity within the fibrocystic liver disease spectrum and frequently co-occurs with Caroli disease, von Meyenburg complex, and autosomal dominant or recessive polycystic kidney disease (ADPKD/ARPKD) (2). Although more than six decades have elapsed since the detailed description by Kerr et al. (3). in 1961, the precise incidence and prevalence of CHF remain elusive, with only several hundred cases reported in the literature to date, suggesting that the true disease burden may be underestimated (2). With the advancement of non-invasive diagnostic modalities such as ultrasonography, computed tomography (CT), and magnetic resonance imaging (MRI), the detection rate of CHF may have increased; however, the substantial clinical heterogeneity of the condition poses considerable challenges to both diagnosis and the assessment of disease burden. In this review, we summarise the pathophysiology, genetics, clinical presentations, diagnostic approaches, management of complications and therapeutic strategies for CHF, as well as the challenges inherent in disease management.

## 2 Search strategy and article selection

A literature search was performed across major databases, including PubMed and Medline. Keywords such as “Congenital hepatic fibrosis”, “Ductal plate malformation”, “Portal hypertension”, “PKHD1 gene”, “Cholangitis”, “Hypersplenism”, “Liver transplantation” and were used in various combinations, with the latest search conducted in June 2026. We used Ryyan (Rayyan Systems Inc., Cambridge, MA, USA; https://www.rayyan.ai/) to screen the studies. We included reviews, metanalyses, clinical trials, observational studies and experimental studies. We identified and screened 4,437 studies. Only studies published in English were included. Additionally, references from relevant articles were screened to identify other pertinent studies not captured in the initial search. Recent articles were selected based on the authors’ judgment regarding their relevance to the topic.

## 3 Pathophysiology and genetics

### 3.1 Pathogenesis and histopathological features of ductal plate malformation

CHF is a rare autosomal recessive disorder caused by defective embryonic ductal plate remodelling, which results in persistent, abnormally dilated primitive ductal structures (2). This anomaly forms a central part of the fibropolycystic liver disease (FLDs) spectrum, together with Caroli disease, polycystic liver disease, and biliary hamartomas, all arising from ductal plate insults at distinct developmental stages: early insults affect large bile ducts (Caroli disease and intrahepatic main duct cysts), intermediate insults affect medium-sized ducts (polycystic liver disease), and late insults affect small ducts (CHF and biliary hamartomas) (4). Histopathologically, CHF is characterised by a diagnostic triad of extensive portal tract fibrosis, increased numbers of morphologically abnormal bile ducts, and preserved hepatic lobular architecture (5). Portal areas are expanded by dense fibrous tissue containing proliferating, irregularly contoured ductules—direct evidence of ongoing ductal plate malformation (6).

### 3.2 Implicated genetic variants and molecular pathways

The pathogenesis of CHF is intimately associated with defective ciliary protein function arising from mutations in multiple genes (7). The genetic landscape of CHF extends well beyond a single gene disorder, encompassing a spectrum of syndromic and non-syndromic forms that share the hallmark of ductal plate malformation and periportal fibrosis. Biallelic mutations in PKHD1, encoding fibrocystin/polyductin, represent the core genetic determinant underlying autosomal recessive polycystic kidney disease with accompanying CHF, while mutations in genes such as ZFYVE19, ANKS6, SEC63, SEC61B, and LRP5 have expanded the understanding of its genetic heterogeneity (7–10). More recently, biallelic variants in KIF12, which encodes a kinesin family motor protein implicated in cholangiocyte ciliary and cytoskeletal function, have been associated with high-gamma-glutamyl transferase (GGT) cholestasis and a spectrum of progressive cholestatic liver disease, further implicating ciliary and cytoskeletal dysfunction in this disease spectrum (11). Additionally, heterozygous pathogenic variants in HNF1B, encoding hepatocyte nuclear factor 1β, may present with neonatal or childhood cholestasis and developmental hepatobiliary abnormalities, frequently as part of a multisystem disorder that includes renal cysts and maturity-onset diabetes of the young (12). Importantly, CHF also occurs as a component of several well-characterised ciliopathy syndromes, including Joubert syndrome (COACH), Meckel syndrome, Jeune syndrome, and Bardet-Biedl syndrome, reflecting the central role of the primary cilium in both biliary and multi-organ development (Table 1).

**Table 1:** Congenital hepatic fibrosis (CHF) and related ciliopathy syndromes: causative genes and characteristic clinical features.

Disease/syndrome	Causative gene(s)	Key clinical features
ARPKD with CHF	PKHD1	Neonatal/infantile markedly enlarged cystic kidneys; congenital hepatic fibrosis with ductal plate malformation; portal hypertension; high perinatal mortality in severe forms; hepatic phenotype may dominate in later-onset cases.
CHF with sclerosing cholangitis and high-GGT cholestasis	ZFYVE19, KIF12	Isolated or predominant hepatic fibrosis accompanied by sclerosing cholangitis; elevated gamma-glutamyl transferase; progressive bile duct injury; renal involvement absent or mild.
Multiorgan ciliopathy with CHF	ANKS6	CHF and renal cystic disease; disrupted Hippo signalling; may also involve heart and skeleton; phenotypic overlap with ARPKD but with broader organ spectrum.
COACH syndrome (Joubert syndrome with hepatic fibrosis)	TMEM67, CC2D2A, RPGRIP1L	Cerebellar vermis hypoplasia; ataxia; intellectual disability; ocular motor apraxia; congenital hepatic fibrosis; renal cysts or nephronophthisis may be present.
Meckel syndrome	MKS1, TMEM67, TMEM216, CC2D2A, CEP290	Occipital encephalocele; postaxial polydactyly; bilateral large cystic kidneys; ductal plate malformation and hepatic fibrosis; usually perinatally lethal.
Jeune syndrome (asphyxiating thoracic dystrophy)	DYNC2H1, IFT80, WDR19, TTC21B	Narrow thorax with short ribs; respiratory distress; renal cystic disease; hepatic fibrosis; retinal degeneration; skeletal dysplasia.
Bardet-Biedl syndrome	BBS1, BBS2, BBS10, and other BBS genes	Obesity; retinitis pigmentosa; postaxial polydactyly; renal anomalies; hypogonadism; hepatic fibrosis occurs in a subset of patients.
CHF associated with endoplasmic reticulum translocation defects	SEC63, SEC61B	Variable severity of CHF; may co-occur with polycystic liver or kidney disease; disrupted ER protein processing.
CHF associated with dysregulated Wnt signalling	LRP5	Fibrocystic liver changes; skeletal abnormalities (e.g., bone fragility); possible vascular and ocular involvement.
HNF1B-associated hepatobiliary disease	HNF1B	Neonatal or childhood cholestasis; developmental hepatobiliary anomalies; often part of a multisystem disorder with renal cysts and maturity-onset diabetes of the young (MODY).

ARPKD, autosomal recessive polycystic kidney disease; GGT, gamma-glutamyl transferase; ER, endoplasmic reticulum.

**Figure 1:**
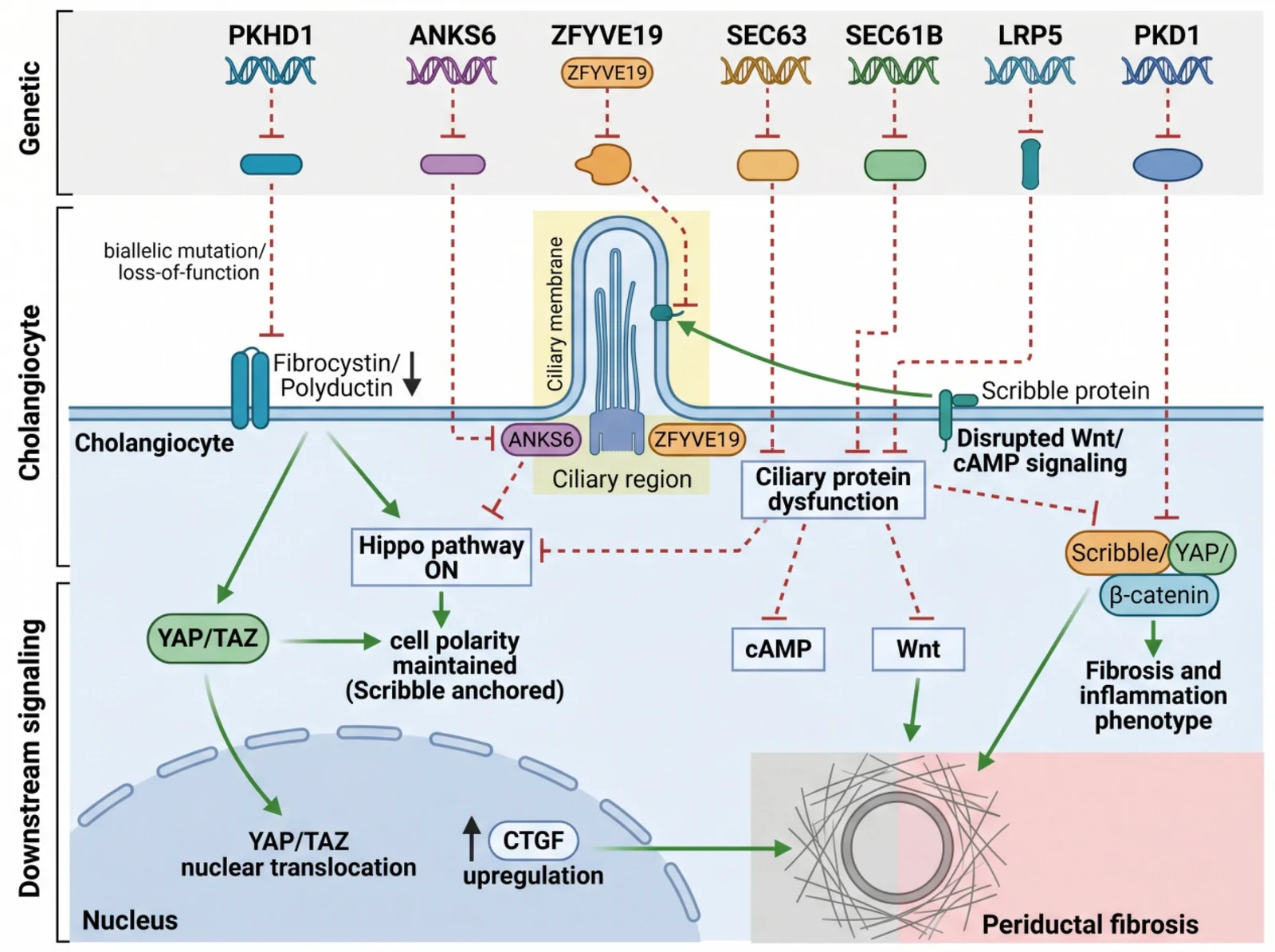
Pathophysiology and associated molecular mechanisms of congenital hepatic fibrosis (CHF). PKHD1 and PKD1 (fibrocystin/polyductin) deficiency leads to Hippo pathway dysregulation, YAP/TAZ activation, CTGF upregulation, and disruption of cAMP and planar cell polarity. ZFYVE19 loss-of-function contributes to ductal plate malformation and cholestasis. ANKS6 loss disrupts Hippo signalling and bile duct development. SEC63 and SEC61B impair endoplasmic reticulum-associated protein translocation, while LRP5 dysregulates Wnt signalling.

At the molecular level, disordered downstream signalling pathways triggered by defective ciliary protein function constitute the central mechanism underlying biliary dysgenesis and progressive fibrosis (13). In cholangiocytes, fibrocystin deficiency leads to dysregulation of the Hippo signalling pathway, promoting YAP/TAZ nuclear translocation and upregulating CTGF expression, thereby directly driving periportal fibrogenesis (13). Loss of ANKS6 function similarly interferes with biliary morphogenesis through antagonism of Hippo signalling (10). Truncating mutations in PKHD1 typically give rise to classical ARPKD (8), whereas certain missense or splice-site variants may manifest as late-onset, predominantly hepatic disease (14). Moreover, rare mutations in genes such as PKD1 can lead to childhood-onset or adult-onset CHF in association with ADPKD (1, 15). Consequently, a comprehensive understanding of genotype–phenotype associations is of substantial clinical importance for disease surveillance, prognostic evaluation and genetic counselling (Figure 1).

## 4 Clinical manifestations and complications

### 4.1 Heterogeneous classification of clinical manifestations

The clinical presentation of CHF exhibits remarkable heterogeneity. Based on the predominant clinical features, CHF is conventionally classified into the portal hypertensive type, the cholangitic type, and the mixed type (16). The portal hypertensive type represents the most common clinical manifestation of CHF, with patients with CHF typically presenting with hepatosplenomegaly and upper gastrointestinal haemorrhage (e.g., haematemesis, melaena) as the initial symptoms (17). Liver function in these patients with CHF is frequently normal or only mildly deranged, which stands in stark contrast to the portal hypertension associated with classic cirrhosis, wherein significant hepatic functional impairment is a prominent feature. The cholangitic type is relatively uncommon; affected individuals chiefly present with recurrent episodes of fever, abdominal pain, and jaundice (18). The underlying pathological basis lies in the concomitant developmental anomaly or dilatation of the intrahepatic bile ducts, which predisposes the patient to secondary bacterial infection. The mixed type encompasses features of both portal hypertension and recurrent cholangitis, thereby rendering the clinical course considerably more complex (16). With regard to age distribution, the portal hypertensive type may be encountered across all age groups but is observed more frequently in children and adolescents, whereas the cholangitic type may be identified at any age following an acute infectious episode.

In addition to the principal subtypes delineated above, CHF is frequently accompanied by several rare yet clinically significant manifestations, of which Caroli syndrome is the most paradigmatic (19). Caroli disease is defined as non-obstructive saccular or fusiform dilatation of the intrahepatic bile ducts; when this entity coexists with CHF, it is designated as Caroli syndrome. This combination substantially elevates the risk of recurrent suppurative cholangitis, intrahepatic lithiasis, and ultimately cholangiocarcinoma. Furthermore, cavernous transformation of the portal vein constitutes another important concomitant finding in CHF, arising from the development of an extensive collateral venous network in response to obstruction of the main portal vein or its branches (20). Other associated manifestations encompass abnormalities in organs that share the same ciliary gene defects (e.g., mutations in TMEM67, WDR19, among others) as CHF, such as retinitis pigmentosa, nephronophthisis, cerebellar vermis hypoplasia and central nervous system malformations (21–23). This underscores the nature of CHF as a systemic disorder, thereby necessitating a comprehensive screening evaluation during clinical assessment.

**Figure 2:**
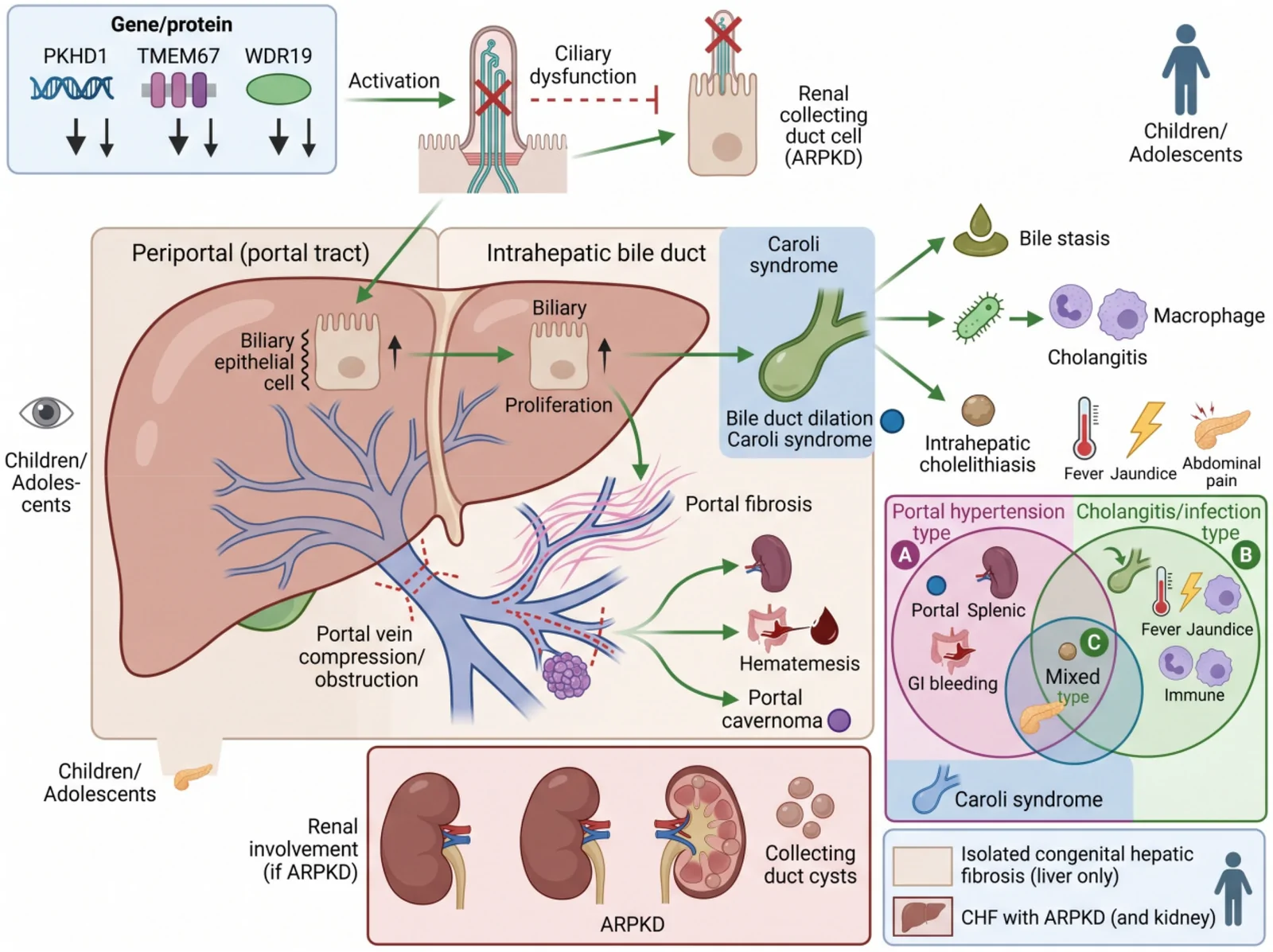
Clinical manifestations and complications of congenital hepatic fibrosis (CHF). Ciliary dysfunction arising from mutations in PKHD1, TMEM67, and WDR19 drives CHF and may involve renal collecting duct cysts in autosomal recessive polycystic kidney disease (ARPKD). Hepatic manifestations include biliary epithelial proliferation and intrahepatic bile duct dilation (Caroli syndrome), leading to cholangitis, intrahepatic choledocholithiasis, and portal hypertension. The latter results in splenomegaly, gastrointestinal bleeding, and portal cavernoma. The condition is clinically stratified into portal hypertension, cholangitis/infection, and mixed subtypes. CHF predominantly presents in children and adolescents.

In the paediatric population, it is important to emphasise that CHF most frequently presents as a component of syndromic ciliopathies rather than as an isolated disorder (21). The distinction between isolated congenital hepatic fibrosis and syndromic forms carries important clinical implications. While isolated CHF typically presents with portal hypertension and its complications in the absence of significant extrahepatic disease, syndromic ciliopathies associated with CHF frequently involve multisystem manifestations, including renal cystic disease, central nervous system abnormalities, retinal dystrophy, and skeletal anomalies (24, 25). These differences in phenotypic spectrum have a substantial impact on clinical presentation, disease course, prognostic counselling, and the need for multidisciplinary surveillance and management. Accordingly, a comprehensive diagnostic evaluation for paediatric patients with CHF should include assessment for extrahepatic involvement to guide appropriate syndromic classification and long-term care planning (Figure 2).

### 4.2 Long-term complications

The sequelae of portal hypertension—oesophagogastric variceal haemorrhage, hypersplenism, and ascites—largely determine prognosis; nearly half of patients with CHF (45.8%) initially present with upper gastrointestinal bleeding, and splenomegaly is found in up to 69.6% of adults (26). Recurrent cholangitis, driven by biliary stasis secondary to ductal plate malformation, is another severe complication, with a cumulative incidence of 45.8% in adult patients with CHF (26, 27). Persistent biliary infection not only accelerates periportal fibrosis and may lead to secondary biliary cirrhosis, reflected by progressive elevation of serum bilirubin, alkaline phosphatase, and gamma-glutamyl transferase, but also creates a chronic inflammatory microenvironment that raises the risk of biliary tract malignancy, particularly cholangiocarcinoma in patients with coexisting Caroli syndrome (7, 27). By contrast, hepatocellular carcinoma is rare in CHF owing to the generally preserved lobular architecture (13, 28). Given these long-term risks, regular imaging surveillance for hepatic space-occupying lesions is warranted, and any unexplained deterioration in liver biochemistry, weight loss, or abdominal pain during follow-up should prompt contrast-enhanced CT or MRI to exclude occult malignancy.

## 5 Diagnostic methods and assessment systems

### 5.1 The core role of diagnostic imaging and technological advances

**Figure 3:**
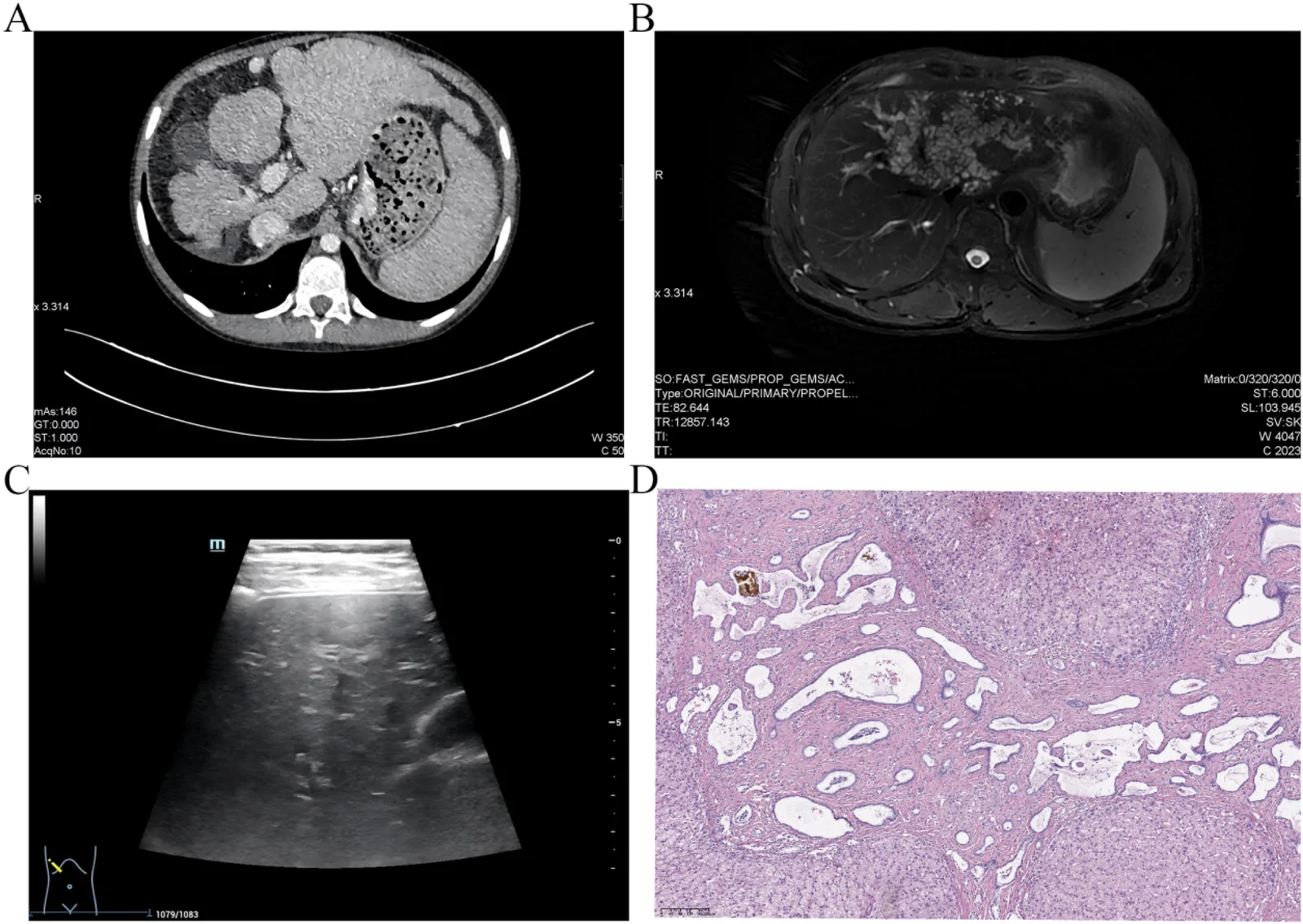
Imaging and pathology features of congenital hepatic fibrosis (CHF). **A**. Abdominal contrast-enhanced CT in CHF may show hepatosplenomegaly, a dysmorphic hepatic contour, dilated intrahepatic bile ducts, features of portal hypertension. **B**. Core MRI findings in CHF include hepatomegaly with a smooth or mildly lobulated contour, T2-hyperintense periportal fibrosis with delayed enhancement, portal hypertension (splenomegaly and collateral vessels), dilated intrahepatic bile ducts. **C**. Abdominal ultrasonography typically shows hepatomegaly with diffusely increased hepatic echogenicity and a coarse echotexture, together with features of portal hypertension, including splenomegaly and portal vein dilatation. **D**. Histopathology (HE 40×) of CHF shows dense periportal fibrosis with numerous irregular, dilated bile duct profiles, consistent with ductal plate malformation. Portal vein branches are often hypoplastic.

Imaging examinations play a central role in the diagnosis, disease assessment, and surveillance of complications in congenital hepatic fibrosis. computed tomography (CT) and magnetic resonance imaging (MRI), particularly magnetic resonance cholangiopancreatography (MRCP), demonstrate considerable superiority in the evaluation of patients with CHF. CT can clearly depict liver morphology, splenomegaly, and the portosystemic collateral circulation associated with portal hypertension, whereas MRI/MRCP, being non-invasive and free of ionising radiation, provides three-dimensional visualisation of the intrahepatic biliary tree anatomy and permits precise assessment of the extent and morphology of biliary dilatation as well as its relationship with the portal vein (Figures 3A,B). This distinction is crucial for differentiating isolated CHF from Caroli syndrome (29).

Ultrasonography, owing to its non-invasive nature, accessibility, and cost-effectiveness, is frequently employed as the first-line screening modality (Figure 3C). Characteristic sonographic findings include diffuse hepatomegaly, diffusely increased echogenicity of the hepatic parenchyma, and periportal fibrotic bands, all of which reflect the underlying pathological changes of portal tract fibrosis typical of CHF (30). Furthermore, ultrasound can effectively identify associated biliary tract abnormalities, such as segmental or saccular dilatations of the intrahepatic bile ducts, which are particularly common in Caroli syndrome (31). Simultaneously, ultrasound serves as the primary method for screening renal lesions that frequently coexist with CHF, such as polycystic kidney disease, and may reveal multiple small cysts in both kidneys. Moreover, these advanced imaging modalities can effectively exclude or diagnose complications such as portal vein thrombosis, cholangitis, and intrahepatic lithiasis, thereby furnishing critical information for clinical decision-making (32).

Transient elastography (FibroScan®) assesses the degree of fibrosis indirectly by measuring liver stiffness and offers the advantages of rapidity and reproducibility (29). Because liver injury in congenital hepatic fibrosis is predominantly presinusoidal, liver stiffness values are often lower than those observed in cirrhosis, particularly during the early stages of the disease. However, stiffness may progressively increase with disease progression and the development of portal hypertension.

In summary, modern ultrasound-based, CT, MR and transient elastography techniques offer valuable non-invasive diagnostic information that should be interpreted in conjunction with clinical and laboratory data. These imaging tools are best regarded as complementary adjuncts that can guide clinical decision-making and, in many cases, reduce the need for invasive sampling. While no single imaging modality currently provides the full histopathological spectrum obtainable by liver biopsy, liver biopsy itself should be reserved for situations in which the diagnosis remains equivocal after comprehensive non-invasive evaluation, or when histopathological confirmation is essential for therapeutic planning or prognostic stratification.

### 5.2 The role of liver biopsy and histopathological diagnostic criteria

Liver biopsy occupies an irreplaceable gold-standard position in the definitive diagnosis of CHF, particularly when the clinical presentation is atypical or when differentiation from other fibrotic liver diseases is required (1). The clinical manifestations of CHF are diverse, ranging from an asymptomatic state to severe complications of portal hypertension, and the condition is frequently confused with viral hepatitis, autoimmune liver disease, and metabolic dysfunction-associated steatotic liver disease (MASLD) (33). Liver biopsy is strongly recommended for histological confirmation when a patient—especially a child or young adult—presents with portal hypertension disproportionate to the degree of hepatic functional impairment, or when imaging reveals concurrent renal cysts or intrahepatic biliary abnormalities (34). Timely histopathological assessment is essential to avoid misdiagnosis as common cirrhosis or haematological disorders and to facilitate the initiation of appropriate management and intervention.

Histopathological evaluation of liver biopsy specimens is central to the diagnosis of CHF, with the identification of characteristic ductal plate malformation and the determination of a specific pattern of fibrosis being the key diagnostic points. Typical histological findings include extensive and dense fibrous tissue proliferation within portal tracts, which forms broad fibrous septa that encircle the hepatic lobules (Figure 3D). Crucially, the lobular architecture itself is generally preserved, and typical pseudolobules—a hallmark of post-hepatitic cirrhosis—are absent, a feature that is critical for distinguishing CHF from cirrhosis (6). Furthermore, immunohistochemical staining, particularly for glutamine synthetase, can provide valuable ancillary diagnostic information (28). In CHF, glutamine synthetase expression is strictly confined to the cytoplasm of hepatocytes located in acinar zone 3. In contrast, in cirrhosis resulting from other aetiologies, the disruption of ductal plate development and the accompanying alterations in hepatic parenchymal architecture may lead to disordered or aberrant expression patterns. This distinction serves as a useful adjunct in differentiating CHF from other causes of liver fibrosis. A meticulous assessment of these histological features, integrated with clinical and radiological findings, is essential for the pathologist to render an accurate diagnosis.

### 5.3 Genetic diagnosis and family screening

With the widespread adoption of high-throughput sequencing technologies, multi-gene panel testing and whole-exome sequencing have become the cornerstone for the definitive diagnosis and differential diagnosis of CHF (35). Because the clinical presentation of CHF frequently overlaps with that of Caroli disease and ARPKD, reliance on clinical and imaging findings alone for differential diagnosis is often problematic (36). The diagnostic workflow generally begins with a comprehensive clinical assessment of the patient suspected of having the condition, followed by multi-gene panel sequencing that includes established causative genes (e.g., PKHD1, ANKS6, WDR19) (10, 21, 37). Should the panel yield a negative result or should there be a high index of suspicion for a novel causative gene, whole-exome sequencing is employed to broaden the scope of detection. This approach not only clarifies the underlying aetiology but also elevates the differential diagnosis of CHF and its related syndromes to the molecular level. Consequently, a systematic genetic diagnostic workflow is an indispensable component for the accurate identification of CHF and its associated disease spectrum.

With respect to genetic counselling, carrier testing of the parents following confirmation of the proband is fundamental for assessing the recurrence risk (38). As CHF is predominantly inherited in an autosomal recessive manner, the parents are typically asymptomatic carriers and the recurrence risk for subsequent offspring is 25%. For high-risk families in whom a definitive molecular diagnosis has been established, prenatal genetic testing is feasible and can provide crucial information to support reproductive decision-making.

**Figure 4:**
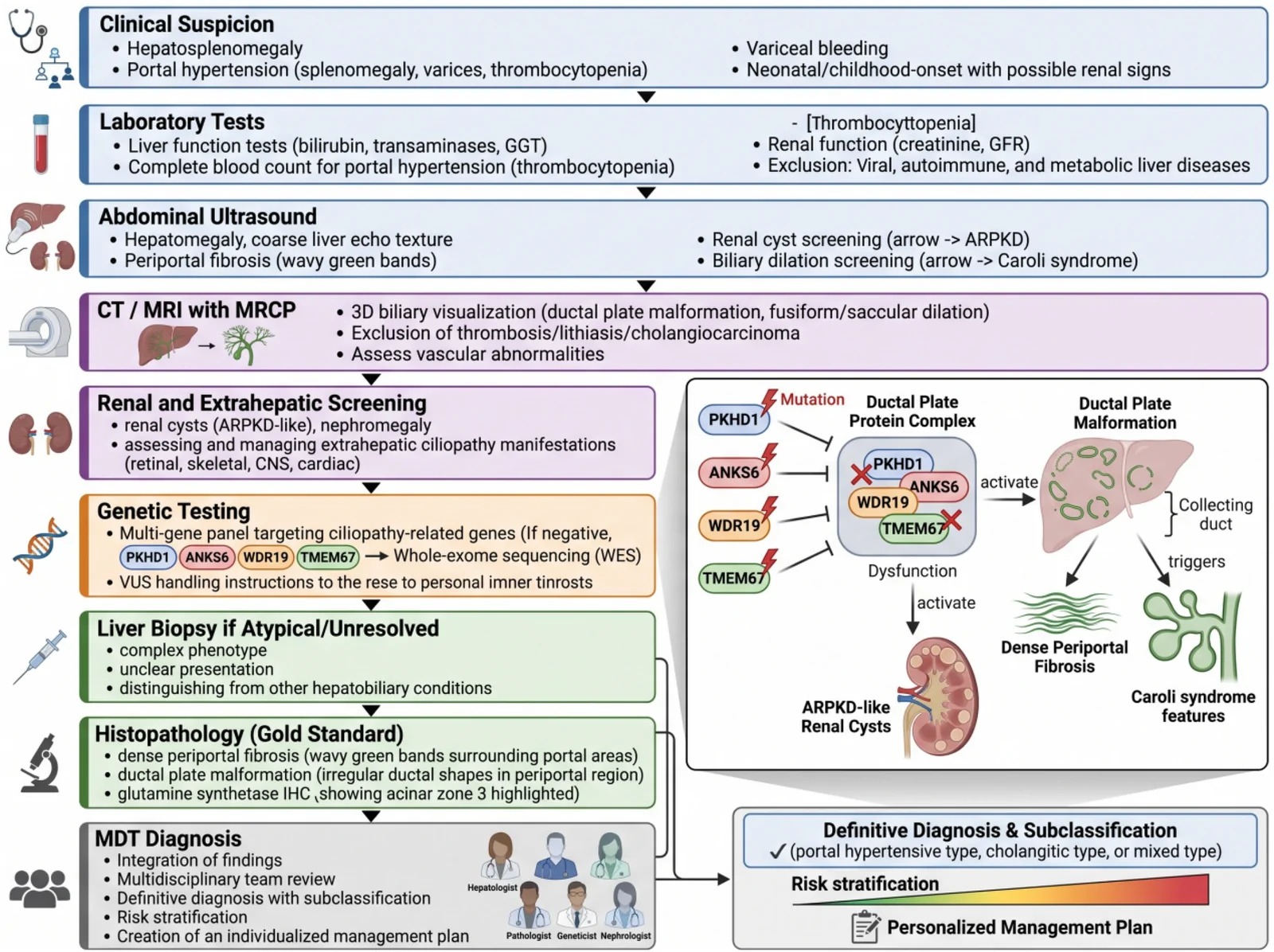
Proposed diagnostic w­orkflow for suspected congenital hepatic fibrosis (CHF). The algorithm begins with clinical suspicion and progresses through laboratory, ultrasonographic, and cross-sectional imaging assessments. Genetic testing employs a ciliopathy-focused multi-gene panel, followed by whole-exome sequencing if required, whilst histopathology remains the gold standard for confirming ductal plate malformation and periportal fibrosis. Definitive diagnosis, subtype classification (portal hypertensive, cholangitic, or mixed), and risk stratification are established via multidisciplinary team consensus, leading to a personalised management plan.

Despite the increasing maturity of genetic diagnostic technologies, several challenges persist in the clinical management of CHF, among which negative results and variants of uncertain significance (VUS) are the most prominent (39). First, even with whole-exome sequencing, a proportion of patients with clinically characteristic CHF remain without an identifiable pathogenic mutation. This may be attributable to the incomplete coverage of disease-associated genes in current knowledge, the inadequate detection of non-coding variants or structural rearrangements, or the existence of genetic mechanisms that have yet to be elucidated. Second, VUS—variants for which the pathogenic potential cannot be classified on the basis of existing evidence—are frequently encountered during testing. Such variants require a comprehensive evaluation that incorporates familial co-segregation analysis, functional studies, allele frequency databases, and phenotype-specific databases. These challenges necessitate close collaboration between clinicians and genetic counsellors to fully explain the limitations of test results to patients with CHF and their families, to avoid the erroneous exclusion of a genetic diagnosis on the basis of a VUS or a negative result, and to underscore the importance of regular clinical follow-up (Figure 4, Table 2).

**Table 2:** Diagnostic features and differential diagnoses of congenital hepatic fibrosis (CHF).

Diagnostic modality	Characteristic findings in CHF	Main differential diagnoses	Key distinguishing features
Clinical presentation	Portal hypertension disproportionate to hepatic functional impairment; onset typically in childhood or young adulthood	Cirrhosis (viral, autoimmune, etc.)	In cirrhosis, portal hypertension parallels hepatic decompensation; in CHF, liver function is long preserved.
Ultrasonography	Diffuse hepatomegaly, increased hepatic echogenicity, periportal fibrotic bands; frequently coexisting renal cysts	ARPKD	ARPKD presents primarily with markedly enlarged, cystic kidneys and renal dysfunction; CHF is liver-dominant.
CT/MRI/MRCP	CT reveals splenomegaly and portosystemic collaterals; MRCP provides three-dimensional biliary tree imaging—segmental or saccular dilatation indicates Caroli syndrome	Caroli disease/Caroli syndrome	Caroli disease has biliary dilatation without periportal fibrosis; Caroli syndrome combines both pathologies.
Liver biopsy (histopathology)	Dense periportal fibrosis with broad fibrous septa; lobular architecture preserved; pseudolobules absent; ductal plate malformation; glutamine synthetase immunohistochemistry strictly confined to acinar zone 3	Cirrhosis (all causes); viral hepatitis; autoimmune hepatitis; non-alcoholic fatty liver disease	Cirrhosis invariably shows pseudolobules and architectural disruption; the other conditions possess distinct serological and histological features.
Genetic testing	Biallelic pathogenic variants in PKHD1, ANKS6, WDR19 and other ciliopathy genes; enables molecular confirmation, genetic counselling and prenatal diagnosis	ARPKD; Caroli disease; other fibrocystic liver-kidney disorders	Substantial genetic overlap (e.g., PKHD1); diagnosis requires clinicopathological integration—ARPKD is renal-dominant, CHF is hepatic-dominant.

ARPKD, autosomal recessive polycystic kidney disease; CT, computed tomography; MRI, magnetic resonance imaging; MRCP, magnetic resonance cholangiopancreatography.

## 6 Management strategies for complications of portal hypertension

### 6.1 Prevention and treatment of oesophagogastric varices (EGV)

EGV are a frequent consequence of portal hypertension in patients with CHF, and their prevention and management are central to clinical care. The Baveno VII consensus provides the current reference framework for the management of portal hypertension, emphasising risk stratification based on clinically significant portal hypertension (CSPH), defined as a hepatic venous pressure gradient (HVPG) ≥ 10 mmHg, and shifting the therapeutic goal from merely preventing variceal bleeding towards the comprehensive prevention of hepatic decompensation (40). Following the diagnosis of CHF, an index upper gastrointestinal endoscopy is advised to screen for varices. In patients with no varices or small (F1) varices, surveillance endoscopy may be performed at extended intervals, with the precise timing informed by dynamic reassessment of liver function, spleen size, platelet count, and hepatic venous pressure gradient where available. For those with medium or large (F2/F3) varices, Baveno VII endorses primary prophylaxis with non-selective beta-blockers (NSBBs), such as propranolol or carvedilol, as first-line therapy, or with endoscopic variceal ligation (EVL) when NSBBs are contraindicated, poorly tolerated, or ineffective (40). In patients with CHF, the frequent coexistence of renal impairment mandates careful monitoring during NSBB therapy.

Acute variceal haemorrhage demands immediate, protocolised resuscitation, including cautious fluid replacement, correction of coagulopathy, and prophylactic broad-spectrum antibiotics, in line with Baveno VII guidance (40). Pharmacotherapy with splanchnic vasoconstrictors—terlipressin being the preferred first-line agent, with somatostatin or octreotide as alternatives—should be initiated promptly to reduce portal pressure. Endoscopy should be performed as soon as possible, ideally within 12 h, to confirm the bleeding source and deliver definitive treatment (41). EVL is the treatment of choice for bleeding oesophageal varices, whereas injection of tissue adhesive (e.g., cyanoacrylate) is recommended for bleeding gastric varices. In patients with uncontrolled haemorrhage despite pharmacological and endoscopic measures, balloon tamponade serves as a temporary bridge to more definitive rescue therapy.

Secondary prophylaxis is of paramount importance to prevent rebleeding. According to Baveno VII, the combination of NSBBs and serial EVL until variceal eradication constitutes the first-line strategy (40). In patients with CHF who experience rebleeding despite optimal combined therapy, the insertion of a transjugular intrahepatic portosystemic shunt (TIPS) offers effective portal decompression. Beyond its role in managing refractory variceal haemorrhage and difficult-to-treat ascites, TIPS can serve as a valuable bridge to liver transplantation in carefully selected candidates, providing temporary haemodynamic stabilisation and control of portal hypertensive complications while the patient awaits a donor organ (40, 42). In the CHF population, pre-existing biliary anatomical abnormalities may increase the technical complexity of TIPS creation and the risk of infectious complications, necessitating meticulous pre-procedural planning. When TIPS is not feasible or has failed, surgical shunting provides an alternative; however, outcomes depend substantially on shunt type. Non-selective shunts divert portal inflow and may impair hepatic function, whereas selective shunts, such as the distal splenorenal shunt, can preserve hepatopetal flow and reduce encephalopathy risk (43). Long-term patency also varies with surgical technique and patient factors. The choice between TIPS and a specific surgical shunt therefore requires comprehensive, multidisciplinary evaluation.

### 6.2 Hypersplenism and the management of ascites

Hypersplenism, manifesting as splenomegaly and peripheral cytopenia—particularly thrombocytopenia—is a common portal hypertensive complication in CHF (44). Mild or asymptomatic cases are managed conservatively with regular blood count monitoring (45). For severe, symptomatic cytopenia refractory to medical therapy, interventional options include splenectomy and partial splenic embolisation (PSE). Splenectomy provides rapid, durable correction of cytopenia but carries risks of overwhelming infection and portal venous thrombosis (46). PSE, a less invasive alternative, effectively elevates platelet and white cell counts while partially preserving splenic immune function, though post-procedural fever, pain, and pleural effusion are common (47, 48). The choice between these procedures requires individualised assessment of hepatic reserve, ascites, coagulation status, and splenic size (49).

Ascites heralds decompensated portal hypertension in CHF and is managed through a stepwise approach (50). Initial therapy consists of dietary sodium restriction (<2 g/day) and diuretics, typically spironolactone alone or combined with furosemide, titrated to achieve a daily weight loss of 0.3–0.5 kg (0.8–1.0 kg if peripheral oedema is present) (51). Large-volume paracentesis with intravenous albumin replacement (6–8 g per L of ascites removed) is indicated for tense or refractory ascites (52, 53). Patients with CHF unresponsive to these measures should be considered for TIPS or liver transplantation (26). Because renal impairment frequently coexists with CHF, diuretic therapy necessitates close monitoring of renal function and electrolytes to prevent hepatorenal syndrome.

Spontaneous bacterial peritonitis (SBP) is a potentially fatal complication of ascites in CHF. High-risk patients with CHF (prior SBP, gastrointestinal haemorrhage, low ascitic fluid protein) benefit from long-term prophylactic quinolones such as norfloxacin (54, 55). Diagnosis relies on an ascitic fluid polymorphonuclear count ≥250 cells/mm^3^; empirical treatment with a third-generation cephalosporin should be started immediately, with a typical duration of 5–7 days (55, 56). A repeat paracentesis at 48 h is performed to assess response. Albumin infusion is used to maintain effective circulating volume and reduce the risk of hepatorenal syndrome. In patients with refractory ascites and recurrent SBP, evaluation for liver transplantation should be pursued as the definitive intervention addressing portal hypertension (57).

## 7 Prevention and management of biliary-related complications

### 7.1 Diagnosis and management of cholangitis

CHF is characterised by extensive ductal plate malformation and bile ductular proliferation, which impair bile drainage and predispose patients to recurrent cholangitis (5). Episodes may present with fever, abdominal pain and jaundice, but are often more frequent and refractory than in the general population, and can occur without a demonstrable biliary obstruction (non-stenotic cholangitis) (58, 59). The Tokyo Guidelines 2018 (TG18) provide a standardised diagnostic and severity-grading framework (60), and emerging biomarkers such as presepsin show promise in predicting severe cholangitis and positive blood cultures (61).

Once acute cholangitis is diagnosed, empirical antibiotics should be started promptly, targeting the predominant biliary pathogens—Gram-negative bacilli (Escherichia coli, Klebsiella pneumoniae) and anaerobes (62, 63). Patients with a history of endoscopic sphincterotomy or biliary stent placement are at elevated risk of Enterococcus infection (64). Standard empirical regimens target Gram-negative organisms and anaerobes; however, third- and fourth-generation cephalosporins lack clinically relevant enterococcal activity, and meropenem is not reliably active against these organisms (65). When Enterococcus coverage is required, ampicillin is the treatment of choice for susceptible Enterococcus faecalis, with vancomycin as an alternative in β-lactam-intolerant patients with CHF or when guided by susceptibility testing (66, 67). For severe illness or suspected resistant pathogens, broader-spectrum agents may be indicated, but enterococcal coverage must be addressed separately (68). Treatment duration, traditionally 7–14 days, may be shortened once adequate biliary drainage is achieved (62, 68–70).

For patients with highly frequent, quality-of-life-compromising episodes despite optimised drainage, prophylactic antibiotics may be considered (71, 72). Given the risks of selecting drug-resistant organisms and disrupting the gut microbiota (73), prophylaxis should be individualised, closely monitored, and preferentially employ a rotating antibiotic strategy (74).

### 7.2 Interventions for biliary stones and strictures

Patients with CHF frequently harbour complex biliary malformations that predispose to stone formation through cholestasis, recurrent cholangitis, and bile duct structural anomalies (75). Stones may cause biliary obstruction, acute cholangitis, and, in the long term, accelerate hepatic fibrosis and potentially increase the risk of cholangiocarcinoma. Management is complicated by the frequent coexistence of multiple strictures, which creates a vicious cycle of “stricture–stone–infection” that impairs bile drainage and promotes recurrence. Endoscopic retrograde cholangiopancreatography (ERCP) with sphincterotomy and basket or balloon extraction remains the first-line approach for common bile duct stones (76). For intrahepatic or large, impacted stones not amenable to conventional ERCP, advanced techniques such as peroral or single-operator cholangioscopy permit lithotripsy under direct visualisation (77, 78); when ERCP is not feasible, percutaneous transhepatic cholangioscopy (PTCS) combined with the SpyGlass™ system offers an alternative (79). Surgical stone extraction is reserved for endoscopic and interventional failures, particularly for stones exceeding 15 mm or complex strictures (80). For benign biliary strictures, endoscopic balloon dilatation provides short-term benefit, but recurrence is common (81). Temporary stent placement is therefore a key strategy for maintaining long-term patency; fully covered self-expanding metal stents (FCSEMS) have shown superior efficacy and removability (82), and modified short FCSEMS combined with a contralateral plastic stent has proven effective for hilar strictures (83). Stent-related complications—migration, in-stent restenosis, and sludge or stone formation—remain clinical challenges (84). For complex, long-segment strictures refractory to endoscopic management, surgical options such as bile duct reconstruction, hepatectomy, or bilioenteric anastomosis should be considered (85). Overall, the management of biliary stones and strictures in CHF requires an individualised, multimodal approach integrating endoscopic, percutaneous, and surgical modalities, with long-term follow-up to detect recurrence.

## 8 Indications and timing of liver transplantation

In patients with CHF, liver transplantation is indicated for refractory complications of portal hypertension, including recurrent variceal haemorrhage, intractable ascites, and recurrent cholangitis. Further indications include secondary liver failure, suspected malignant transformation—particularly the elevated risk of cholangiocarcinoma in Caroli syndrome—and hepatopulmonary syndrome, for which early transplantation is warranted even when liver function is relatively preserved (86–89). The assessment of transplant timing is especially complex in children and adolescents, requiring a careful balance between the risks of disease progression and those of surgery and lifelong immunosuppression; decisions should integrate the control of portal hypertensive complications, frequency and severity of cholangitis, nutritional and growth status, and quality of life (8, 86).

When CHF coexists with ARPKD, combined liver-kidney transplantation is indicated for concurrent end-stage liver and kidney disease (90–92). In patients with severe portal hypertension but relatively preserved renal function, sequential transplantation—liver first, followed by kidney transplantation later if necessary—may be appropriate, based on comprehensive evaluation of the patient's overall condition and available resources (93, 94) (Table 3).

**Table 3:** Management strategies for complications of congenital hepatic fibrosis and corresponding levels of evidence.

Complication	Management strategy	Specific intervention(s)	Level of evidence	Comments
Oesophagogastric varices	Primary prophylaxis	NSBBs (propranolol, carvedilol) first-line; EVL if NSBBs contraindicated or not tolerated	B	Extrapolated from cirrhosis data and Baveno VII; monitor renal function in CHF
	Acute variceal haemorrhage	Resuscitation, vasoconstrictors (terlipressin preferred), antibiotics, urgent endoscopy with EVL (oesophageal) or cyanoacrylate (gastric); balloon tamponade as bridge	B	Follows Baveno VII; no CHF-specific trials
	Secondary prophylaxis	NSBB + serial EVL (first-line); TIPS for refractory rebleeding or as bridge to transplant; surgical shunt (e.g., distal splenorenal) if TIPS not feasible or fails	B (combination therapy) C (TIPS/surgery)	TIPS/surgery evidence from case series; biliary anomalies increase TIPS complexity
Hypersplenism	Conservative management	Observation and monitoring of blood counts	C	For asymptomatic or mild cytopenia.
	Pharmacological therapy	Thrombopoietin receptor agonists	D	Limited specific experience in CHF.
	Interventional/surgical therapy	Partial splenic embolisation (PSE); splenectomy	C	PSE preserves some splenic immune function; splenectomy carries risks of infection and thrombosis.
Ascites	Stepwise medical management	Dietary sodium restriction; aldosterone antagonists (spironolactone) ± loop diuretics (furosemide)	B	Close monitoring of renal function and electrolytes is mandatory.
	Large-volume ascites	Therapeutic paracentesis with intravenous albumin replacement	B	Albumin at 6–8 g per L of ascites removed to prevent post-paracentesis circulatory dysfunction.
	Refractory ascites	TIPS; evaluation for liver transplantation	B	TIPS may be complicated by hepatic encephalopathy.
Spontaneous bacterial peritonitis (SBP)	Prevention	Long-term oral quinolones (norfloxacin) for high-risk patients	A	Targeted at those with prior SBP, gastrointestinal haemorrhage, or low ascitic fluid protein.
	Diagnosis and treatment	Diagnostic paracentesis (PMN ≥ 250 cells/mm^3^); third-generation cephalosporins for 5–7 days	A	Empirical antibiotics should be commenced immediately; repeat paracentesis at 48 h to assess response.
Cholangitis	Diagnosis and severity grading	Tokyo Guidelines 2018 (TG18) criteria; emerging biomarkers (presepsin)	B	Integrate systemic inflammation, cholestasis, and imaging findings.
	Empirical antimicrobial therapy	Cover Gram-negative bacilli and anaerobes; add ampicillin (or vancomycin) for enterococci if prior sphincterotomy or stent. Avoid reliance on cephalosporins or meropenem alone for enterococcal coverage.	B (standard), C (short-course)	Ensure dedicated enterococcal coverage when risk factors present. Short-course therapy is safe following source control.
	Prophylaxis for recurrent episodes	Long-term or rotating antibiotics	C	Reserve for highly frequent episodes after optimising biliary drainage; monitor for resistance.
Biliary stones and strictures	First-line intervention	ERCP with sphincterotomy and stone extraction; balloon dilatation for strictures	B	Complex anatomy may limit success.
	Advanced endoscopic techniques	Peroral cholangioscopy (POC) with lithotripsy; fully covered self-expanding metal stent (FCSEMS) for strictures	C	For intrahepatic or impacted stones and refractory strictures.
	Percutaneous/surgical options	Percutaneous transhepatic cholangioscopy (PTCS); surgical stone extraction or bilioenteric anastomosis	C	Reserved for endoscopic failure.
Liver transplantation	Indications	Refractory variceal haemorrhage, recurrent cholangitis, refractory ascites, secondary liver failure, suspected malignancy, hepatopulmonary syndrome	B	Indications are mainly based on severity of portal hypertensive and biliary complications rather than classical liver failure.
	Timing and combined transplantation	Combined liver-kidney transplantation for CHF with ARPKD; sequential transplantation considered on an individual basis	C	Requires multidisciplinary assessment of overall disease burden and organ function

Levels of evidence: A: Consistent evidence from high-quality randomised controlled trials or systematic reviews; B: Evidence from well-designed cohort or case-control studies, or extrapolated from high-quality studies in similar populations; C: Evidence from case series, retrospective studies, or expert consensus with a strong clinical rationale; D: Expert opinion or limited anecdotal experience. EVL, endoscopic variceal ligation; TIPS, transjugular intrahepatic portosystemic shunt; PSE, partial splenic embolisation; SBP, spontaneous bacterial peritonitis; PMN, polymorphonuclear; TG18, Tokyo Guidelines 2018; ERCP, endoscopic retrograde cholangiopancreatography; POC, peroral cholangioscopy; FCSEMS, fully covered self-expanding metal stent; PTCS, percutaneous transhepatic cholangioscopy; ARPKD, autosomal recessive polycystic kidney disease.

## 9 Comprehensive assessment of disease burden

### 9.1 Patient-reported outcomes and their impact on quality of life

CHF, as a chronic and progressive genetic disorder, exerts a disease burden that extends beyond hepatic pathological alterations to profoundly affect patients’ quality of life across physical, psychological, personal and social domains. Although specific instruments for assessing quality of life in CHF are currently lacking, insights from studies of other chronic liver diseases and chronic conditions with serious complications can reveal the broad challenges likely faced by patients with CHF (95). With respect to physical functioning, patients with CHF frequently experience marked fatigue and weakness due to hypersplenism and recurrent gastrointestinal bleeding caused by portal hypertension (1). Concomitant renal disease may additionally cause pain and discomfort, further diminishing health-related quality of life (HRQoL) (96). At the psychological level, the demands of long-term disease management, fear of complications and uncertainty about future health status can readily precipitate anxiety and depression (97). A comprehensive evaluation of the multidimensional impact of CHF on quality of life is therefore a cornerstone for developing patient-centred, integrated management strategies.

The paediatric and adolescent CHF population faces distinct challenges that differ from those in adults, spanning the critical periods of growth and development and exerting a lasting influence on long-term outcomes. Growth retardation is one of the most prominent issues (97). Impaired gastrointestinal absorption due to portal hypertension, recurrent infections, and metabolic disturbances associated with coexisting renal insufficiency can lead to faltering height and weight gain. Such growth impairment not only affects physical development but may also be linked to cognitive development, particularly when comorbid conditions such as congenital hypothyroidism are present (14). Body image concerns are especially pronounced in adolescent patients with CHF. Abdominal distension resulting from marked splenomegaly, skin changes owing to jaundice or pharmacotherapy, and physical limitations that preclude participation in peer sporting activities can undermine adolescents’ body image and self-esteem, intensifying social isolation and emotional distress. Treatment adherence represents another major challenge in this age group. Management of CHF typically entails complex medication regimens, strict dietary restrictions and regular medical monitoring. During adolescence, characterised by a drive for independence and a propensity for rebelliousness, prolonged and burdensome treatment protocols can readily provoke resistance, leading to missed doses or non-adherence to medical advice, which in turn heightens the risk of serious complications such as acute cholangitis and gastrointestinal bleeding. Furthermore, the disease substantially disrupts education (35). Frequent school absences, hospitalisations and disease-related fatigue make it difficult for affected children to keep pace academically, potentially influencing their future educational and vocational choices. These unique challenges necessitate that healthcare teams adopt individualised, family-engaged management strategies and prioritise psychosocial support for both patients with CHF and their families, thereby helping them to better cope with the disease and promoting healthy physical and psychological development.

### 9.2 Socioeconomic burden and healthcare resource utilisation

CHF, a rare hereditary hepatobiliary disease, imposes a socioeconomic burden and requires healthcare resource utilisation that are key dimensions for assessing its overall impact. Although dedicated quantitative studies on the lifetime medical costs of patients with CHF are currently lacking, its economic burden can be reasonably inferred by analysing the disease spectrum and clinical management pathways. The clinical management of CHF extends throughout a patient's life, from infancy and childhood through to adulthood. As the disease progresses, patients with CHF frequently require hospitalisation for various complications and receive treatment; the management of these acute events constitutes a substantial portion of direct medical costs. For patients with CHF who need liver transplantation, the surgical expenses, long-term costs of postoperative immunosuppressive medications, and lifelong follow-up expenditure are extremely high. Research has shown that 30-day readmission in patients with heart failure significantly increases the burden on healthcare systems, with each readmission incurring an average additional cost of US$16,380 and 6.6 additional hospital days (98). Although this study was not conducted in CHF, the pattern it reveals—whereby chronic, complex diseases lead to recurrent hospitalisations and high resource consumption—also applies to the management of CHF, indicating the need for a systematic evaluation of its direct medical costs.

The indirect burden imposed by CHF on patients’ families is equally substantial and is frequently underestimated. Because the disease often manifests in childhood and demands lifelong management, family caregivers must invest considerable time and effort. Caregiving activities include accompanying patients with CHF to frequent hospital follow-up visits, assisting with daily medication, monitoring for and responding to acute complications, and providing bedside support during hospitalisations. These sustained caregiving demands may prevent the primary caregiver from engaging in full-time employment or necessitate a reduction in working hours, leading to a significant loss of household income. This financial strain is even more pronounced for families of children with severe disease or those requiring liver transplantation. Furthermore, the psychological distress associated with the disease, uncertainty about the future, and the travel and accommodation costs incurred when seeking specialist diagnosis and treatment away from home further intensify the family's financial and emotional burden. Although such indirect costs are difficult to monetise precisely, they are an indispensable component of the total disease burden, affecting the quality of life and socioeconomic status of the entire family.

Across different healthcare systems, patients with CHF face significant challenges in obtaining equitable and accessible specialist care. As a rare disease, its diagnosis and treatment are highly dependent on experienced hepatobiliary centres, imaging specialists, and genetic counselling teams. However, the uneven distribution of medical resources may mean that patients with CHF in remote or economically underdeveloped regions struggle to obtain a timely and accurate diagnosis. With regard to treatment, access to liver transplantation is constrained not only by organ donor availability but also by the scope of health insurance coverage and the family's financial capacity. This highlights the importance of establishing standardised clinical pathways and cost-effectiveness evaluation frameworks for rare diseases such as CHF, in order to promote the equitable allocation of healthcare resources and to guide health insurance policy-making. Such measures would help ensure that all patients with CHF, irrespective of their socioeconomic background or geographic location, can receive the necessary, high-quality medical care.

## 10. Challenges and unmet needs in current clinical practice

### 10.1 Issues in diagnostic delay and misdiagnosis

CHF is a rare autosomal recessive disorder for which diagnostic delay and misdiagnosis pose a particularly prominent problem in clinical practice. The prevalence of CHF is extremely low, reported to be approximately 1 in 10,000 to 1 in 20,000, and the condition is even less common in adults (28). The primary reason for diagnostic delay lies in the heterogeneous and non-specific nature of its clinical presentations (6). Patients with CHF most often present with symptoms related to portal hypertension, whilst their hepatocellular function typically remains normal or only mildly impaired. This feature means that when encountering a patient with a cirrhotic-appearing liver, clinicians are highly likely to consider more common aetiologies first—such as viral hepatitis or alcoholic liver disease—thereby overlooking the possibility of CHF (1). Moreover, when a patient exhibits concurrent hepatic and renal abnormalities, insufficient awareness of the association with this ciliopathy may lead to an erroneous diagnostic trajectory or to attention being confined to a single organ (15).

Raising awareness of CHF among non-hepatology specialists is equally critical, because patients with CHF may initially present to various specialties with symptoms originating from different organ systems. Paediatricians may encounter children referred for unexplained hepatosplenomegaly, growth retardation or recurrent biliary tract infections; nephrologists may be the first to identify concomitant polycystic kidney disease or renal impairment; and general practitioners or gastroenterologists are frequently faced with patients with CHF whose initial manifestation is gastrointestinal bleeding or ascites. Strengthening interdisciplinary education to familiarise non-hepatology specialists with the full clinical spectrum of CHF is therefore of paramount importance. Only by heightening the index of suspicion for this disorder across specialties can early and accurate diagnosis be promoted, unnecessary investigations and treatment delays be avoided, and more rational long-term management strategies be formulated for patients with CHF.

### 10.2 Lack of disease-modifying therapies

Currently, all clinical interventions for CHF are symptomatic and supportive, aimed at managing its complications (7). These treatments, however, do not address the root cause of the disease—namely, aberrant bile duct development and fibrosis driven by genetic mutations. Consequently, there is an urgent need in clinical practice for specific disease-modifying therapies (DMTs) that target this process, with the aim of delaying or even halting disease progression and improving long-term patient outcomes.

Based on a deeper understanding of CHF pathogenesis, preclinical studies targeting potential molecular pathways are being explored. Thus, restoring ciliary function or modulating their downstream signalling pathways represents a promising therapeutic direction. Notably, the cyclic adenosine monophosphate (cAMP) signalling pathway plays a key role in cholangiocyte homeostasis and cystic disease; modulators of this pathway have demonstrated efficacy in ADPKD, providing a rationale for investigating their application in CHF (15). In addition, interventions targeting pro-fibrotic pathways such as transforming growth factor-β (TGF-β), inflammatory responses, and cellular senescence are potential strategies (99). However, translating these pathogenesis-based discoveries into clinical therapies faces multiple obstacles. First, CHF is a rare disease with a small patient population, making large-scale clinical trials difficult to conduct. Second, the disease exhibits considerable heterogeneity, with marked variability in clinical manifestations, rates of progression, and comorbidities, which hampers uniform assessment of treatment response. Third, the lack of animal models that accurately recapitulate human CHF disease progression limits thorough preclinical pharmacodynamic validation. Finally, the therapeutic window for CHF may be exceedingly narrow; how to intervene early in the disease course, or even prenatally, poses a major challenge for future translational medicine. In conclusion, the development of disease-modifying therapies for CHF remains an arduous task, demanding close collaboration among basic research, translational medicine, and clinical practice.

### 10.3 Gaps in long-term follow-up and multidisciplinary management

Currently, the long-term follow-up management of CHF faces significant challenges. The core problem lies in the absence of unified evidence-based follow-up guidelines, which has led to marked disparities in the monitoring parameters and frequency adopted in clinical practice. CHF is a disease with considerable clinical heterogeneity; patients with CHF can progress from asymptomatic hepatosplenomegaly to severe complications (1). Nevertheless, there is no internationally recognised consensus on when surveillance should begin, which indices should be monitored, or what the optimal monitoring intervals should be. This lack of standardised monitoring means that some patients with CHF may miss the window for intervention owing to insufficient follow-up, while others may be subjected to unnecessary burdens from excessively frequent examinations. Moreover, CHF frequently co-exists with renal disease, which means that long-term follow-up must address both hepatic and renal function, further increasing the complexity of developing comprehensive follow-up guidelines. Therefore, there is an urgent need to establish large patient cohorts and to conduct long-term prospective studies to delineate the key events and risk factors in the natural history of the disease, thereby providing a robust evidence base for formulating standardised, individualised long-term follow-up protocols.

Establishing a truly effective multidisciplinary team (MDT) collaborative model is essential for the holistic management of patients with CHF but encounters numerous practical difficulties. The diagnosis and treatment of CHF involve multiple specialties, including hepatology, radiology, pathology, clinical genetics, general surgery, endoscopy, nutrition, and psychology. Real-world obstacles to achieving such efficient collaboration include poor interdisciplinary communication, the absence of a unified patient management platform, uneven distribution of healthcare resources, and diagnostic delays stemming from insufficient awareness of this rare disease. Consequently, promoting a patient-centred, institutionalised CHF-MDT model and utilising digital tools to share patient data are pivotal steps towards raising the overall standard of care and improving long-term patient outcomes.

The inadequacy of patient registries for congenital hepatic fibrosis severely constrains in-depth investigation of the disease's natural history and the conduct of clinical trials. Because CHF is a rare disease, any single centre accumulates only a limited number of cases, making it difficult to perform large-scale, convincing clinical studies. Currently, there is no unified, prospective CHF patient registry worldwide, which results in an incomplete understanding of the long-term disease trajectory from childhood to adulthood, the true effectiveness of various interventions, and the key prognostic factors. This fragmented data landscape hinders the accurate evaluation of new diagnostic and therapeutic strategies and also impedes the design of clinical trials and patient recruitment for novel drug development targeting CHF. Establishing an international or national CHF patient registry, with standardised collection of clinical, imaging, pathological, genetic, and long-term follow-up data, would not only provide a more comprehensive picture of the full disease spectrum but also serve as an invaluable resource for identifying distinct clinical subtypes, validating biomarkers, and conducting multicentre clinical trials. Only through systematic data accumulation and sharing can the research bottleneck imposed by the rarity of the disease be overcome, ultimately driving substantive progress in the diagnosis and treatment of CHF.

## 11. Future research directions and perspectives

### 11.1 Frontiers in basic and translational research

In recent years, significant advances in basic and translational research have provided important directions for deepening our understanding of disease mechanisms and developing novel therapies. First, the use of patient-derived cells for disease modelling is crucial for investigating the pathological mechanisms of congenital hepatic fibrosis (CHF). Although the direct generation of biliary organoid models from CHF patient-derived induced pluripotent stem cells (iPSCs) has not yet been reported in the literature, related studies have offered important insights that support this approach. A Pkhd1 knockout mouse model has been established and successfully recapitulates the pathological features of CHF, including biliary abnormalities and rapidly progressive periportal fibrosis (10). Furthermore, transcriptomic analysis of the PCK rat—a model of CHF/ARPKD—has uncovered alterations in gene expression profiles associated with cell migration, adhesion and wound healing, providing candidate targets for validating specific pathways in more refined organoid models (100). In the future, integrating patient-specific iPSCs with organoid technology holds promise for generating *in vitro* models that more closely mirror the physiological state of the human disease, enabling high-throughput screening of compounds capable of correcting biliary developmental defects or inhibiting fibrosis and thereby accelerating therapeutic discovery (101).

Gene therapy, particularly gene editing technologies such as CRISPR/Cas9, offers long-term hope for fundamentally correcting the specific gene mutations that underlie CHF. Researchers have successfully employed CRISPR/Cas9 to create a CHF mouse model carrying a Pkhd1 mutation (Pkhd1^del3–4/del3–4^), which not only reproduced the hepatic fibrosis phenotype but also, through multi-omics analyses, revealed distinct gut microbial and metabolite signatures, providing new perspectives on the full spectrum of the disease (102). This demonstrates the powerful capacity of gene editing to generate precise disease models. Nevertheless, translating gene editing into clinical application still faces considerable technical bottlenecks. The primary challenge is a safe and efficient *in vivo* delivery system that can precisely target editing tools to liver cells while avoiding off-target effects and immunogenicity. Second, the genotype–phenotype relationship in CHF is complex, and different mutations within the same gene can lead to varying disease severity, necessitating highly individualised and precise gene therapy strategies. Moreover, in patients with CHF with advanced disease and extensive fibrosis, simply correcting the genetic mutation may be insufficient to reverse established architectural damage and must be combined with anti-fibrotic strategies. Despite these immense challenges, the rapid progress in gene editing technology paints a tantalising blueprint for achieving curative treatment of CHF in the future.

Novel anti-fibrotic strategies that target the fibrotic microenvironment have shown considerable promise in the treatment of CHF. At the core of liver fibrosis lie the activation of hepatic stellate cells (HSCs) and excessive extracellular matrix deposition, processes that are finely orchestrated by a complex microenvironment involving immune cells (103, 104). Studies have demonstrated that macrophages are key drivers of the fibrotic process, and that oxytocin can act on hepatic macrophages to promote their shift from a pro-inflammatory Ly6C^hi^ phenotype to a reparative Ly6C^low^ phenotype, thereby reversing liver fibrosis (105). These findings underscore the potential of immunomodulation as a new therapeutic target in CHF. In addition, “redox biology” strategies that address the disrupted redox balance within the fibrotic microenvironment, as well as plant extracts with natural anti-fibrotic activities, have also exhibited therapeutic potential (106, 107). Emerging extracellular vesicle (EV)-based therapies are particularly compelling: EVs, as natural intercellular communication carriers, can be harnessed as precision tools for the targeted delivery of anti-fibrotic agents to activated HSCs, offering innovative approaches to restore hepatic microenvironment homeostasis (108). These multi-dimensional strategies targeting the fibrotic microenvironment are expected to complement gene therapy and together form the core of a future comprehensive therapeutic system for CHF.

### 11.2 Clinical research and practice optimisation directions

CHF is a rare disease, and advancing clinical research and optimising practice are essential to improving patient outcomes. Currently, the core challenges in the field include a paucity of natural history data, a lack of effective targeted therapies and inadequate assessment tools. Therefore, future research and practice should focus on systematic data collection, the design of targeted clinical trials, and the establishment of a patient-centred outcome assessment framework. Given the rarity of CHF, a single centre struggles to accumulate a sufficient number of cases to conduct robust and convincing research. Hence, establishing international patient registries and biobanks is an urgent priority. Such platforms can integrate global case information, including detailed clinical phenotypes, genotypes, imaging and pathological data, as well as long-term follow-up data. Through large-scale, standardised data collection, the natural history of the disease can be delineated more accurately, and the critical factors influencing disease progression and prognosis can be identified.

Beyond traditional pathological and biochemical markers, a comprehensive assessment of treatment benefit in CHF must incorporate patients’ subjective experience and quality of life. At present, there is a lack of validated, CHF-specific QoL assessment instruments and patient-reported outcome (PRO) measures. Generic liver disease QoL scales may fail to capture the unique challenges faced by patients with CHF. Therefore, the development and validation of CHF-specific QoL/PRO instruments represents an important direction for clinical research. This process should adhere to rigorous instrument development guidelines, with qualitative interviews used to gain an in-depth understanding of the illness experiences of patients with CHF and their caregivers, identifying the symptoms, aspects of functional status and psychological well-being domains that matter most to patients with CHF, thereby generating an item pool. Subsequently, reliability, validity and responsiveness should be evaluated through large-scale international multicentre studies. Such tools can not only measure, with greater sensitivity, the improvement in quality of life conferred by new therapies—providing crucial evidence for drug regulatory approval and health technology assessment—but also help clinicians to understand more comprehensively the disease burden and treatment needs of patients with CHF in everyday clinical practice, thus achieving truly patient-centred care.

## 12. Conclusion

CHF, a chronic liver disease driven by ductal plate malformation, has long centred on the symptomatic management of portal hypertension and biliary complications (6). Although current strategies have achieved considerable success in controlling most complications, they remain essentially supportive and do not address the underlying disease cause, leaving patients with CHF with a substantial burden of disease progression risk, recurrent hospitalisations and diminished quality of life. The core challenges facing the current diagnostic and therapeutic framework include diagnostic delay, the absence of disease-modifying therapies capable of altering the disease course, and the fragmentation of long-term follow-up and patient support systems. These unmet needs underscore the urgency of a fundamental strategic shift from “treating established disease” towards “preventing disease” or “delaying its progression”.

Future directions must rest on the synergistic advancement of multiple pillars. The foremost priority is to deepen basic research, leveraging cutting-edge technologies such as genomics and organoid models to precisely dissect the molecular mechanisms underlying ductal plate malformation, thereby identifying druggable targets and laying the groundwork for genuine disease-modifying therapies. Secondly, clinical study design requires optimisation, with an emphasis on prospective, multicentre collaboration and the establishment of an international congenital hepatic fibrosis patient registry. Such a registry would not only provide real-world natural history data but also enable efficient patient recruitment into clinical trials, accelerating the evaluation of novel therapies. When reconciling differing research perspectives, it is vital to recognise the irreplaceable value of supportive symptomatic treatment at the present stage, whilst actively channelling research resources towards causal therapies, thus achieving an integration of short-term symptom control with long-term disease-modifying goals.

The ultimate vision is to construct a patient-centred, evidence-based, individualised and comprehensive management paradigm. This demands close collaboration among multidisciplinary teams encompassing hepatology, radiology, pathology, surgery, nutrition and psychology. Treatment goals should no longer be confined to “firefighting” after complications emerge; instead, efforts should be devoted to delaying or even halting hepatic fibrosis progression early in the disease course through surveillance, risk stratification and potential novel interventions. Realising this ambition depends on sustained basic scientific breakthroughs, innovative clinical trials, a well-developed healthcare system and robust patient community support, thereby truly improving the long-term outcomes and quality of life of individuals with congenital hepatic fibrosis (Table 4).

**Table 4:** Take-home messages.

Take-home messages
CHF is a rare autosomal recessive ciliopathy characterised by ductal plate malformation and periportal fibrosis, leading to non-cirrhotic portal hypertension with preserved liver function, predominantly in children and young adults.
Clinical presentation is markedly heterogeneous; a disproportionate severity of portal hypertension relative to hepatic impairment should prompt specific diagnostic evaluation.
Definitive diagnosis relies on integrated imaging (ultrasound, MRCP), characteristic liver histopathology (ductal plate malformation, absence of pseudolobules) and genetic testing (e.g., PKHD1), alongside systematic screening for renal and other ciliopathy-associated organ involvement.
Current management remains largely symptomatic, centred on the prevention and treatment of variceal haemorrhage, cholangitis and other portal hypertensive complications; disease-modifying therapies are conspicuously absent.
Major unmet needs include the lack of standardised long-term follow-up protocols, fragmented multidisciplinary care, absence of CHF-specific patient-reported outcome instruments and inadequate patient registries, all of which hinder clinical research and optimal care delivery.
Future progress requires international collaborative registries, development of targeted anti-fibrotic and gene therapies, and implementation of individualised, multidisciplinary care models that address the unique medical and psychosocial challenges of paediatric and adolescent patients.

CHF, congenital hepatic fibrosis; MRCP, magnetic resonance cholangiopancreatography.
